# A longitudinal study of the relationship between receptivity to e-cigarette advertisements and e-cigarette use among baseline non-users of cigarettes and e-cigarettes, United States

**DOI:** 10.1186/s12971-017-0145-8

**Published:** 2017-11-06

**Authors:** Israel T. Agaku, Kevin Davis, Deesha Patel, Paul Shafer, Shanna Cox, William Ridgeway, Brian A. King

**Affiliations:** 1 Office on Smoking and Health, National Center for Chronic Disease Prevention and Health Promotion, Centers for Disease Control and Prevention, 4770 Buford Hwy NE, Mailstop F-79, Atlanta, GA 30341 USA; 2 RTI International, Atlanta, GA USA

**Keywords:** E-cigarettes, Advertisements, Tobacco control, Policy, Receptivity, Initiation

## Abstract

**Background:**

We investigated the relationship between receptivity to electronic cigarette (e-cigarette) advertisements at baseline and e-cigarette use at follow-up among adult baseline non-users of cigarettes and e-cigarettes.

**Methods:**

A nationally representative online panel was used to survey non-users of cigarettes and e-cigarettes (*n* = 2191) at baseline and 5-month follow-up. At baseline, respondents were shown an e-cigarette advertisement and asked if they were aware of it (exposure). Among those exposed, receptivity was self-rated for each ad using a validated scale of 1 to 5 for agreement with each of six items: “worth remembering,” “grabbed my attention,” “powerful,” “informative,” “meaningful,” and “convincing.” Logistic regression was used to measure the relationship between receptivity at baseline and e-cigarette use at follow-up.

**Results:**

Among baseline non-users of cigarettes and e-cigarettes, 16.6% reported exposure to e-cigarette advertisements at baseline; overall mean receptivity score was 2.77. Among baseline non-users who reported exposure to e-cigarette advertisements, incidence of e-cigarette use at follow-up was 2.7%; among baseline non-users who reported not being exposed to e-cigarette advertisements, incidence of e-cigarette use at follow-up was 1.3%. The attributable risk percentage for e-cigarette initiation from e-cigarette advertisement exposure was 59.3%; the population attributable risk percentage from e-cigarette advertisement exposure was 22.6%. Receptivity at baseline was associated with e-cigarette use at follow-up (aOR = 1.57; 95% CI = 1.04–2.37).

**Conclusions:**

Receptivity to e-cigarette advertisements at baseline was associated with greater odds of e-cigarette use at follow-up among baseline non-users of cigarettes and e-cigarettes. Understanding the role of advertising in e-cigarette initiation could help inform public health policy.

## Background

Electronic cigarette (e-cigarette) advertising expenditures in the United States increased approximately 18-fold from 2011 ($6.4 million) to 2014 ($115 million) [1, 2]. Correspondingly, U.S. e-cigarette sales have increased rapidly in recent years, reaching $2.5 billion in 2014 [3, 4]. Some e-cigarette advertisements have included claims of relative advantages of e-cigarettes over conventional cigarettes, including that e-cigarettes are healthier, more socially acceptable, or could be used to quit conventional cigarette smoking [5, 6]. An estimated 58.4% of current cigarette smokers who use e-cigarettes report doing so to quit conventional cigarette smoking [7], despite inconclusive evidence on the efficacy of e-cigarettes for long-term cessation [8].

Several cross-sectional studies have demonstrated an association between e-cigarette advertisement exposure and actual or intended e-cigarette use among adults [6, 9, 10]. However, these cross-sectional studies are limited by the inability to establish temporality between exposure and outcome. Further information on the impact of e-cigarette advertising exposure on use could help inform regulatory efforts to prevent e-cigarette initiation and established use, especially among youth and young adults [11, 12]. Therefore, this longitudinal study investigated the relationship between receptivity to e-cigarette advertisements and current e-cigarette use among a national sample of U.S. adults who were baseline non-users of conventional cigarettes and e-cigarettes.

## Methods

### Data

We used data from a nationally representative longitudinal online survey of US adults aged ≥18 years administered by GfK Custom Research. Participants were recruited from a probability sample of residential postal addresses covering approximately 95% of all U.S. households. Invitation letters were mailed to all sampled households and contained website links and passwords to enable the selected household to access the survey. The probability of selection was known for all participants and participants could not volunteer for study enrollment. Those who were not Internet-enabled were provided additional study incentive payments to complete the survey in public locations with Internet access, such as libraries.

The survey was conducted in two waves: April 12 to June 30, 2014 (baseline) and September 11 to November 17, 2014 (follow-up). Non cigarette smokers were defined as respondents who never smoked or who reported smoking at least 100 cigarettes in their lifetime, but smoked “not at all” at baseline. Non e-cigarette users were persons who reported that they used e-cigarettes “not at all” at baseline. All baseline non users of cigarettes or e-cigarettes who participated at baseline (*n* = 3123) were re-contacted for follow-up approximately 5 months later; a longitudinal retention rate of 74.6% was achieved. All analyses reported in this study are based on the longitudinal cohort of *n* = 2191 persons who neither smoked cigarettes nor used e-cigarettes at baseline and who completed both survey waves.

### Measures

#### Exposure to e-cigarette advertisements at baseline

To measure exposure to e-cigarette advertisements, respondents were shown one of 5 popular e-cigarette advertisements (three Blu and two Njoy advertisements) at random via a video stream within the survey. Those unable to view the video stream were shown a storyboard of images from the advertisement. Using this protocol to cue recall, participants were then asked to indicate whether they had seen the e-cigarette advertisement on either television or online in the past 3 months. Respondents who reported having seen an advertisement in the past 3 months were defined as having being exposed to the e-cigarette advertisement they viewed.

#### Receptivity to e-cigarette advertisements at baseline

Receptivity to e-cigarette advertisements among those who reported being exposed was measured with a multi-item scale similar to those used in previous research [13]. After viewing each advertisement in the survey, each respondent was asked whether he or she agreed or disagreed with the following statements: (1) “this ad was worth remembering”; (2) “this ad grabbed my attention”; (3) “this ad was powerful”; (4) “this ad was informative”; (5) “this ad was meaningful”; and (6) “this ad was convincing”. Each item was assessed on a scale from 1 (*strongly disagree*) to 5 (*strongly agree*). Item-specific responses were averaged for each advertisement, and then averaged across advertisements, to obtain a single value (range 1–5).

#### Smoking history and awareness of tips advertisements

Cigarette smoking history of baseline non-users of cigarettes and e-cigarettes was explored using a lifetime threshold of 100 cigarettes; respondents were classified as never smokers (smoked <100 cigarettes in lifetime) or former smokers (smoked ≥100 cigarettes in a lifetime but were not smokers at the time of the survey).

The 2014 wave of the Centers for Disease Control and Prevention’s national tobacco education campaign *Tips From Former Smokers* (*Tips*) aired in two 9-week phases that overlapped with the study period (Phase 1: February 3–April 6, 2014; Phase 2: July 7–September 7, 2014) [14]. Therefore, we assessed exposure to *Tips* advertisements (“yes” or “no”) as a potential confounder.

#### Current e-cigarette use at follow-up

Current e-cigarette use at follow-up was defined as using e-cigarettes “some days” or “every day” (vs. “not at all”).

### Statistical analysis

Subgroup differences in exposure and receptivity were assessed using χ2 and Wald tests. Based on prevalence of e-cigarette use at Wave 2 by advertisement exposure at Wave 1 among baseline non-users of cigarettes and e-cigarettes, we estimated the attributable risk percentage (among those exposed) and the population attributable risk percentage (among the entire population).

Multivariable logistic regression was used to measure the association between receptivity to e-cigarette advertisements and e-cigarette use at follow-up among baseline non-users of cigarettes and e-cigarettes, controlling for sex, age, race/ethnicity, awareness of *Tips* advertisements, cigarette smoking history, educational attainment, and presence of a smoker in the household. We controlled for regional variation in e-cigarette consumption by including region fixed effects. Data were weighted, and corresponding population totals were calculated for select estimates; statistical significance was ascertained using a threshold of *p* < 0.05.

## Results

Table 1 summarizes characteristics of study participants at baseline. A majority of respondents were non-Hispanic white (69.4%), male (52.4%), and ages 25 to 64 (68.8%). About one-third (34.5%) had attained at least a college degree, and over two-third (68.9%) were never smokers.

**Table 1:** Baseline Exposure^a^ and Receptivity^b^ to E-cigarette Advertisements and E-Cigarette Use^c^ at Follow-Up among Baseline Non-users of Cigarettes and E-cigarettes (*n* = 2191)

	Distribution		Exposure^a^ to E-cigarette Advertisements at Baseline			Mean Receptivity^b^ to E-cigarette Advertisements at Baseline	
Demographic variable	%	N	Prevalence [95% CI]	*P*-Value (χ2 test)	Weighted Population Count [95% CI], millions	Mean Scale Score [95% CI]	*P*-Value (ANOVA)
All nonsmokers	100.0	2191	16.6 (14.7–18.5)	–	33,914,032	2.77 (2.72–2.83)	–
Age, years							
18–24	11.3	264	11.4 (6.2–16.7)	0.241	2,639,467	2.88 (2.71–3.05)	0.027
25–44	33.3	776	17.4 (13.9–21)		11,854,580	2.67 (2.58–2.76)	
45–64	35.5	828	17.2 (14.1–20.4)		12,483,029	2.79 (2.7–2.87)	
65+	19.8	462	17.2 (13.1–21.2)		6,937,030	2.86 (2.76–2.96)	
Sex							
Male	52.4	1221	15.6 (13–18.1)	0.265	16,658,131	2.77 (2.7–2.84)	0.849
Female	47.6	1110	17.8 (14.9–20.6)		17,256,086	2.78 (2.7–2.85)	
Race/ethnicity							
White, non-Hispanic	69.4	1617	14.5 (12.6–16.5)	0.009	20,577,407	2.68 (2.63–2.73)	<0.001
Black, non-Hispanic	10.3	239	28.1 (20.1–36.1)		5,880,073	3.01 (2.81–3.21)	
Hispanic	7.1	166	16.3 (8.3–24.3)		2,374,098	2.69 (2.49–2.88)	
Other, non-Hispanic	13.3	309	18.8 (12.2–25.4)		5,086,565	3.15 (2.96–3.34)	
Education							
< High school	9.5	221	22.2 (13.9–30.4)	0.002	4,293,227	3.23 (3.01–3.44)	<0.001
High school	26.8	624	16.6 (12.8–20.5)		9,078,229	2.86 (2.75–2.97)	
Some college	29.3	683	19.9 (16.3–23.6)		11,909,948	2.77 (2.69–2.86)	
≥ College degree	34.5	803	12.3 (9.8–14.8)		8,633,227	2.58 (2.51–2.65)	
Cigarette smoking history							
Never smokers	68.9	1605	16.0 (13.6–18.3)	0.301	22,418,717	2.8 (2.73–2.86)	0.170
Former smokers	31.1	726	18.1 (14.8–21.4)		11,495,304	2.72 (2.64–2.81)	
Household smoking							
No smoker in household	88.6	2064	15.5 (13.5–17.4)	0.006	27,946,577	2.76 (2.70–2.81)	0.118
Smoker in household	11.4	267	25.4 (18.6–32.1)		5,924,750	2.9 (2.73–3.08)	

*Abbreviations: CI* confidence interval, *e-cigarette* Electronic cigarette

^a^Exposure, a binary variable (yes or no) was assessed at baseline by showing respondents an e-cigarette advertisement selected randomly from 5 popular TV and online advertisements and asking if they were aware of it

^b^Receptivity was computed as an average of six items, each item self-rated on a scale from 1 (*strongly disagree*) to 5 (*strongly agree*) describing the perceived effectiveness of the advertisement shown to the respondent. The six items measured in relation to the advertisement’s effectiveness were “worth remembering,” “grabbed my attention,” “powerful,” “informative,” “meaningful,” or “convincing.” Responses were averaged for each ad and then across advertisements to obtain a single value for a respondents’ overall receptivity of the e-cigarette advertisements

^c^Current e-cigarette users at follow-up were defined as persons who reported using e-cigarettes some days or every day

### Exposure to E-cigarette advertisements at baseline

Overall, 16.6% of nonsmoking U.S. adults (33.9 million) were exposed to an e-cigarette advertisement at baseline. By race/ethnicity, prevalence of self-reported exposure to an e-cigarette advertisement was highest among non-Hispanic blacks (28.1%) and lowest among non-Hispanic whites (14.5%; *p* = 0.009). By education, prevalence of exposure was highest among those with less than a high school education (22.2%) and lowest among those with at least a college degree (12.3%; *p* = 0.002). Prevalence was significantly higher among those who lived with a smoker in the household (25.4%) compared to those who did not (15.5%). No significant differences in e-cigarette advertisement exposure was observed by age or sex (see Table 1).

### Receptivity to E-cigarette advertisements at baseline

The overall mean receptivity score among baseline non-users was 2.77. By age, the mean score was highest among those aged 18–24 years (2.88) and lowest among those aged 25–44 years (2.67) (*p* = 0.027). By race/ethnicity, mean receptivity scores were highest among those classified as ‘other, non-Hispanic’ (3.15) and lowest among non-Hispanic whites (2.68) (*p* < 0.0001). By education level, mean receptivity scores were highest among those with less than a high school education (3.23) and lowest among those with at least a college degree (2.58) (*p* < 0.0001). No significant gender differences were noted for receptivity to e-cigarette advertisements.

### Incidence and determinants of current E-cigarette use at follow-up

Among all baseline non-users of cigarettes and e-cigarettes, 1.3% (2.7 million persons) reported current e-cigarette use at follow-up (Table 2). Among baseline non-users who reported exposure to an e-cigarette advertisement at baseline, 2.7% reported e-cigarette use at follow-up; among baseline non-users who reported not being exposed to an e-cigarette advertisement at baseline, 1.1% reported e-cigarette use at follow-up. In relation to e-cigarette initiation, the attributable risk percentage due to e-cigarette advertisement exposure was 59.3%, and the population attributable risk percentage was 22.6%.

Demographic differences in incidence of e-cigarette use among baseline non-users of cigarettes and e-cigarettes were observed. By race/ethnicity, incidence was highest among non-Hispanic whites (1.6%) and lowest among non-Hispanic blacks (0.3%) (*p* = 0.029). By education, incidence was highest among those with only a high school education (2.1%) and lowest among those with a college degree or higher (0.3%) (*p* = 0.004). Incidence of e-cigarette use at follow-up also varied significantly by smoking history and presence of another smoker in the household. The follow-up incidence among former smokers was 2.5% compared with 0.8% among never smokers (*p* = 0.011). By household smoking, incidence was 0.7% at follow-up among those with no smoker in the household and 6.1% among those with a smoker in the household (*p* = 0.004). No significant differences were noted by age or sex.

Receptivity to e-cigarette advertisements at baseline among non-users of cigarettes and e-cigarettes was significantly associated with e-cigarette use at follow-up (aOR =1.57; 95% CI = 1.04–2.37) (Table 3). Among baseline non-users, the odds of e-cigarette uptake at follow-up were lower among males than females (aOR = 0.35; 95% CI = 0.14–0.90). Former smoking (aOR = 4.30; 95% CI = 1.47–12.61) and presence of another smoker in the household (aOR = 6.48; 95% CI = 2.47–16.97) predicted e-cigarette use at follow-up. Baseline age, awareness of *Tips* advertisements, race/ethnicity, and education were not significantly associated with e-cigarette use at follow-up.

**Table 2:** Incidence of e-cigarette initiation among Baseline Non-users of Cigarettes and E-cigarettes, by e-cigarette advertising exposure status (*n* = 2191)

	Incidence of Current E-cigarette Use at Follow-up (Overall)			Incidence of Current E-cigarette Use at Follow-up (Aware of E-Cig Ads at Wave 1)			Incidence of Current E-cigarette Use at Follow-up (Not Aware of E-Cig Ads at Wave 1)		
Demographic Variable	Percentage	(95% CI)	Weighted Population Count	Percentage	[95% CI)	Weighted Population Count	Percentage	(95% CI)	Weighted Population Count [95% CI]
All Nonsmokers	1.3%	[0.8–1.9]	2,691,273	2.7%	[0.7–4.6]	905,369	1.1%	[0.5–1.6]	1,796,599
Age, years									
18–24	2.1%	[0.0–4.3]	485,112	8.5%	[0.0–19.9]	223,162	1.3%	[0–3.2]	261,949
25–44	1.5%	[0.5–2.5]	1,007,386	4.5%	[0.0–9.2]	529,714	0.9%	[0.3–1.5]	479,273
45–64	0.8%	[0.2–1.5]	613,341	0.7%	[0.0–1.6]	83,690	0.9%	[0.1–1.6]	531,337
65+	1.4%	[0.0–2.9]	585,452	0.9%	[0.0–2.8]	65,417	1.6%	[0–3.3]	525,335
Sex									
Female	1.8%	[0.9–2.7]	1,929,369	3.9%	[0.7–7.1]	650,465	1.4%	[0.5–2.3]	1,289,194
Male	0.8%	[0.2–1.4]	761,808	1.5%	[0.0–3.8]	256,953	0.6%	[0.2–1.1]	506,934
Race/ethnicity									
White, non-Hispanic	1.6%	[0.9–2.3]	2,234,571	2.7%	[0.3–5.1]	546,425	1.4%	[0.7–2.1]	1,690,657
Black, non-Hispanic	0.3%	[0.0–0.8]	53,746	0.9%	[0.0–2.8]	55,330	0.0%	N/A	N/A
Hispanic	0.7%	[0.0–2.1]	103,884	4.4%	[0.0–12.9]	103,884	0.0%	N/A	N/A
Other, non-Hispanic	1.1%	[0.0–2.6]	299,244	3.9%	[0.0–11.3]	196,937	0.5%	[0–1.1]	103,048
Education									
< High school	1.3%	[0.0–2.8]	255,772	2.4%	[0.0–7.2]	103,879	1.0%	[0–2.3]	151,893
High school	2.1%	[0.6–3.5]	1,132,051	3.1%	[0.0–7.6]	280,627	1.9%	[0.4–3.4]	859,112
Some college	1.9%	[0.7–3.1]	1,108,118	3.7%	[0.0–7.6]	443,413	1.4%	[0.3–2.5]	670,965
≥ College degree	0.3%	[0.0–0.5]	195,436	0.9%	[0.0–2.4]	78,196	0.2%	[0–0.4]	117,260
Cigarette smoking history									
Never smokers	0.8%	[0.2–1.3]	1,072,102	1.4%	[0.0–3.4]	318,581	0.6%	[0.1–1.2]	758,379
Former smokers	2.5%	[1.3–3.8]	1,619,151	5.1%	[0.9–9.3]	584,683	2.0%	[0.8–3.2]	1,038,552
Household smoking									
No smoker in HH	0.7%	[0.3–1.1]	1,273,343	0.8%	[0.0–1.8]	227,183	0.7%	[0.3–1.1]	1,046,171
Smoker in HH	6.1%	[2.5–9.7]	1,427,945	11.4%	[1.6–21.2]	677,053	4.3%	[0.8–7.8]	750,893

Note: Source for Cigarette Smoking Prevalence Estimate among all US adults aged 18 years and older during 2014 was the National Health Information Survey, available at http://www.cdc.gov/mmwr/preview/mmwrhtml/mm6444a2.htm?s_cid=mm6444a2_w. Source for population projection for US adults aged 18 years and older during 2014 was the U.S. Census, available at https://www.census.gov/population/projections/data/national/2014/downloadablefiles.html

**Table 3:** Odds Ratios for Current E-cigarette Use^a^ at Follow-up among Baseline Non-users of Cigarettes and E-cigarettes (*n* = 2191)

Characteristic	aOR	95% CI
Receptivity to e-cigarette advertisement at Baseline^b^	1.57*	[1.04,2.37]
Aware of Tips advertisement at Baseline	0.61	[0.23,1.57]
Gender (reference: female)		
Male	0.35*	[0.14,0.90]
Age (reference: 18–24)		
25–44	0.98	[0.23,4.16]
45–64	0.32	[0.07,1.47]
65+	0.44	[0.06,3.11]
Race/ethnicity (reference: white)		
Black	0.20	[0.02,1.58]
Hispanic	0.72	[0.18,2.88]
Other	0.53	[0.09,3.13]
Education (reference: < high school)		
High school	1.57	[0.37,6.66]
Some college	1.34	[0.30,6.05]
≥ College degree	0.32	[0.06,1.59]
Cigarette smoking history (reference: never smoker)		
Former smoker	4.30*	[1.47,12.61]
Household smoking (reference: no household smoker)		
Someone else in household smokes	6.48*	[2.47,16.97]

Note**:** Model controls for region fixed effects

*Abbreviations: AOR* Adjusted odds ratio, *CI* confidence interval, *e-cigarette* Electronic cigarette

*p < 0.05

^a^Current e-cigarette users at follow-up were defined as persons who reported using e-cigarettes some days or every day

^b^Receptivity was computed as an average of six items, each item self-rated on a scale of 1 to 5 (from 1 *strongly disagree*, to 5 *strongly agree*) describing the perceived effectiveness of the advertisement shown to the respondent. The six items measured in relation to the advertisement’s effectiveness were: “worth remembering”; “grabbed my attention”; “powerful”; “informative”; “meaningful” or “convincing.” Responses were averaged for each ad and then across advertisements to obtain a single value for a respondents’ overall receptivity of the e-cigarette advertisements

## Discussion

Approximately 1 in 6 U.S. adults who did not smoke conventional cigarettes reported exposure to an e-cigarette advertisement at baseline. Among baseline non-users of cigarettes and e-cigarettes, receptivity to e-cigarette advertisements at baseline was associated with higher odds of using e-cigarettes at follow-up. These findings suggest that the responsible regulation of e-cigarette advertising targeted at vulnerable populations may be warranted to minimize potential public health harms. For example, restrictions can be placed on media where e-cigarettes can be advertised in an effort to prevent e-cigarette initiation and established use among susceptible populations, particularly youth and nonsmoking adults. To better monitor tobacco marketing activities over time, e-cigarette companies could also be required to report to the Federal Trade Commission their annual advertising and promotional expenditures, overall and by advertising channel, as is currently required for cigarettes and smokeless tobacco products [15, 16].

In May 2016, the U.S. Food and Drug Administration finalized a rule extending its authority to all tobacco products, including e-cigarettes and enables future rulemaking regarding tobacco product manufacturing, marketing, and sales [17]. Given the rapidly evolving and expanding e-cigarette market, efforts are also warranted at the state, local, and tribal government levels to address e-cigarette marketing, advertising, and sponsorship activities that may appeal to non-users of any tobacco product, particularly vulnerable populations, such as youth and young adults.

We found differences among sociodemographic groups in baseline exposure and receptivity to e-cigarette advertisements; specifically, racial/ethnic minorities and persons with lower education reported higher exposure and receptivity to e-cigarette advertisements. These differences could be due, in part, to industry targeting of lower socioeconomic groups. Not all e-cigarette advertising is from major tobacco companies, but the tobacco industry comprises a large segment of the e-cigarette market share and has a history of targeting racial/ethnic minorities with conventional tobacco product promotional activities and advertisements [18, 19].

This study’s major strength is the use of longitudinal data to assess the effect of receptivity to e-cigarette advertisement on e-cigarette initiation. Nonetheless, there are some limitations to this study. First, tobacco use status was self-reported and may have been subject to misreporting. Second, we were unable to measure exposure to all existing e-cigarette advertisements and may thus have underestimated prevalence of exposure to e-cigarette advertisements. Because of space constraints in the survey, each participant was only shown one advertisement selected randomly from a set of several existing advertisements. This is therefore not a measure of overall awareness to the entire spectrum of e-cigarette advertisements featured on different channels, including TV, the internet, magazines, and other print and non-print media. Nonetheless, even with the conservative estimation of exposure, prevalence of exposure (16.6%) was relatively high, and significant associations between receptivity to e-cigarette advertisements at baseline and current e-cigarette use at follow-up were observed, thus emphasizing the reach and impact of e-cigarette advertisements. Fourth, the survey did not collect data on history of e-cigarette use; thus, never and former users could not be differentiated in the analysis. Finally, given the relatively low initiation rate (1.1%), there was large variability in some point estimates, as indicated by wide confidence intervals.

## Conclusion

Among adult non-users of e-cigarettes and conventional cigarettes at baseline, receptivity to e-cigarette advertisements was associated with higher odds of using e-cigarettes at follow-up. These findings underscore the importance of efforts to address e-cigarette advertising, promotion, and sponsorship activities that may lead to initiation of e-cigarette use by nonsmokers.

## Abbreviations

E-cigarettes: Electronic cigarettes
OR: Odds ratios
